# Mental health treatment programs for children and young people in secure settings: A systematic review

**DOI:** 10.1186/s13033-023-00599-2

**Published:** 2023-10-12

**Authors:** Valerie Schutte, Evangeline Danseco, Gabrielle Lucente, Purnima Sundar

**Affiliations:** Knowledge Institute on Child and Youth Mental Health and Addictions, 695 Industrial Avenue, Ottawa, ON K1G 0ZI Canada

**Keywords:** Mental health, Treatment, Secure, Child, Risk to self, Risk to others

## Abstract

**Background:**

While there are mental health treatment programs for children and young people in secure settings (i.e., secure treatment programs) in many countries, there is a lack of transparency and consistency across these that causes confusion for stakeholders and challenges for the design and delivery of high-quality, evidence-based programs. This systematic review addresses two questions: What do mental health treatment programs for children and young people in secure community settings look like across jurisdictions? What is the evidence underlying the various components of these programs?

**Methods:**

Twelve databases were searched in November 2021: CINAHL, EMBASE, MEDLINE, PsycINFO, PubMed, Scopus, Science Direct, Academic Search Complete, Psychology and Behavioral Sciences Collection, Google Scholar, OpenDOAR, and GreyLit.org. To be included, publications had to be empirical literature or a report on mental health treatment within a secure setting for people under the age of 25; contain pre-identified keywords; be based on a research or evaluation study conducted since 2000; and be assessed as low risk of bias using an adaptation of the Critical Appraisal Skills Programme qualitative research checklist. The systematic review included 63 publications. Data were collected and analyzed in NVivo qualitative software using a coding framework.

**Results:**

There are secure treatment programs in Australia, Belgium, Canada, New Zealand, the Netherlands, England and Wales, Scotland, and the United States. Although there are inconsistencies across programs in terms of the systems in which they are embedded, client profiles, treatments provided, and lengths of stays, most share commonalities in their governance, definitions, designs, and intended outcomes.

**Conclusions:**

The commonalities across secure treatment programs appear to stem from them being designed around a need for treatment that includes a mental disorder, symptom severity and salience involving significant risk of harm to self and/or others, and a proportionality of the risks and benefits of treatment. Most share a common logic; however, the evidence suggested that this logic may not to lead to sustained outcomes. Policymakers, service providers, and researchers could use the offered recommendations to ensure the provision of high-quality secure treatment programming to children and young people with serious and complex mental health needs.

**Supplementary Information:**

The online version contains supplementary material available at 10.1186/s13033-023-00599-2.

## Background

Mental health treatment programs in secure settings (i.e., secure treatment programs) are often used when no other service has the capacity to safely manage and address the complex needs of children and young people who have serious mental health concerns and are at high risk of harming themselves and/or others [1–6]. There is no universal program definition, neither is there a consistent term used to refer to these programs: numerous terms are used, and the same term can refer to different programs [7]. Generally, this type of program provides compulsory stabilization and treatment to children when the risk that their mental health concerns pose to self or others has been demonstrated to an authority [7]. While there are secure treatment programs in many countries around the world, there is a lack of transparency, consistency, and stability across secure treatment programs that can hinder the provision of quality services [7–9]. There are large variations in program designs and delivery that can cause confusion among stakeholders and researchers [7–9]. Moreover, the systems in which secure treatment programs are embedded commonly change, and professionals in positions to refer children and young people to secure treatment may not be aware of the programs [9]. These can be barriers to access, collaboration, and coordination [8–11].

There are concerns about the quality of secure treatment programs [8, 12–14]. Little is known about their clinical outcomes [15]. The literature raises questions about their effectiveness [10, 11, 15–22], efficiency and timeliness [4, 5, 9], and equity [4, 8, 9, 23–25]. Quality processes are limited by a lack of consistent data within and across programs [9, 14, 26].

Children and young people deserve timely access to the best mental health treatment experiences and a system that is easy to navigate [27]. Secure treatment programs must be of the highest quality and in the best interest of the children and young people treated in them [28]. There is a pressing need to create a common understanding of what secure treatment programs are and what the evidence about them is to inform consistent, coherent, coordinated, and evidence-based mental health treatment for children and young people in secure settings. In this paper, we present an overview of what secure treatment programs are in different jurisdictions, offer a definition of secure treatment that draws on the commonalities across these programs, and synthesize the evidence about the components of secure treatment programs.

The purpose of this systematic review was to collate and synthesize studies pertaining to the mental health treatment of children and young people in secure community-based mental health settings to address the research questions:

1. What do mental health treatment programs for children and young people in secure settings look like across jurisdictions?
2. What is the evidence underlying the various components of mental health treatment programs for children and young people in secure settings?

The systematic review focuses on mental health treatment in secure community-based mental health settings and not mental health treatment in secure hospital, juvenile justice, and child welfare settings. This is because the systematic review was commissioned by the Ministry of Health of the province of Ontario in Canada to learn the evidence about secure treatment in similar jurisdictions to inform the development of a framework for secure treatment in Ontario. Moreover, in Ontario and other Canadian jurisdictions, secure treatment is a community-based mental health service provided in health centres rather than hospitals [29].

## Methods

### Eligibility criteria

Publications had to meet eligibility criteria (Additional file 1). To be included, a publication had to be empirical literature or a descriptive or evaluative report; in English and/or French^1^; on the subject of mental health treatment for children and/or young people^2^ in a secure setting; based on research or an evaluation conducted in a high-income country and published since 2000; and contain at least one term from each of the three search concepts: secure setting, mental health and addictions treatment, and children and young people. Publications were excluded if they met any of the exclusion criteria: a publication other than empirical literature or a descriptive or evaluative report; based on research or an evaluation conducted in a country with an income level classification other than high-income; in a language other than English or French; on a subject other than mental health treatment; for a population other than children and/or young people; and/or without a term from each of the search concepts or using the terms in a way that does not refer to mental health and addictions treatment for children and young people in a secure setting (e.g., “secure treatment” as the verb “to secure”).

### Information sources

In November 2021, 12 databases were searched: CINAHL, EMBASE, MEDLINE, PsycINFO, PubMed, Scopus, Science Direct, Academic Search Complete, Psychology and Behavioral Sciences Collection, Google Scholar, OpenDOAR, and GreyLit.org. Additional searches sourced documents referenced in a literature review by the Ontario Ministry of Health on secure treatment service delivery models. In March 2022, reference lists of included publications were searched.

### Search strategy

A search strategy focused on three concepts – secure setting, mental health and addictions treatment, and children and young people – was used in English and French (Table 1). The first concept was focused on the secure aspect of secure treatment because this term is used to refer to this type of treatment in Commonwealth countries. As the systematic review was commissioned to inform the development of a framework in Canada, a Commonwealth country, this helped focus the search to similar jurisdictions. Identified citations were imported into Endnote.

### Selection process

Titles and abstracts of identified publications were imported into Covidence (https://www.covidence.org), which identified and removed duplicates. These were screened for keywords by two reviewers independently. The two reviewers trained on screening and full text review by reviewing the criteria together, practicing applying the criteria together to the same five records, and then practicing applying the criteria to five records separately and comparing interrater reliability. To be screened in, titles and abstracts had to include at least one key term from each of the three concepts. Interrater reliability was 87.34% agreement and 0.58 (moderate) Cohen’s kappa.

**Table 1:** *Search strategy*

Term between search terms	Concept 1: Secure treatment	AND	Concept 2: Mental health and addictions	AND	Concept 3: Children and young people
	Secure treatment		Mental health		Youth
OR	Secure care		Mental illness*		Child*
OR	Secure residential treatment		Mental disorder*		Young people
OR	Secure accommodation		Psychiatric illness*		Young person*
OR	Secure facilit*		Addict*		Adolescen*
OR	Secure residential youth care				Young adult*
OR	Secure services				
OR	Secure mental health setting				
OR	Secure setting				

Full texts were screened by two reviewers independently using the eligibility criteria. To be included, both reviewers had to agree that a publication met all the inclusion criteria. To be excluded, both reviewers had to agree that a publication failed to meet one or more of the inclusion criteria and/or met one or more of the exclusion criteria. Interrater reliability was 78.87% agreement and 0.58 (moderate) Cohen’s kappa. Where the two reviewers disagreed, they discussed their justifications. If they did not reach consensus, a third reviewer reviewed the full text and determined its eligibility.

Texts were then assessed for risk of bias using an adaptation of the Critical Appraisal Skills Programme [30] qualitative research checklist. The traditional checklist assesses three broad issues – Are the results of the study valid? What are the results? Will the results help locally? – through 10 questions that can be answered “yes/not applicable”, “can’t tell”, or “no” [30]. The first two questions are screening questions about whether there is a clear research statement and an appropriate methodology [30]. The eight detailed questions address the appropriateness of the research design, the recruitment strategy, and data collection; considerations given to ethics and relationships between researchers and participants; the rigour of analysis; the clarity of the statement of findings; and the value of the research [30]. In terms of the adaptation, a third screening question was added – “The language does not clearly indicate bias” – due to the highly political nature of secure treatment [7] and the bias evident in publications reviewed when piloting the search strategy (e.g., using language biased against the clients of secure treatment, such as describing them using derogatory terms; using language that indicates causation where it should indicate correlation; stating that the purpose of the research article is persuasive). Two reviewers assessed the risk of bias of each publication independently before undertaking consensus together. To be included, publications had to be assessed as minimal risk of bias (i.e., eight or more questions answered as “yes”). Publications assessed as greater than minimal risk were excluded (i.e., less than eight questions answered as “yes”).

### Data collection process

A jurisdiction-based approach to data collection was used, which involved each of the three reviewers collecting data from one or more jurisdiction, to support understanding of the contexts in which secure treatment programs operate. The data items in Additional file 2 were coded and collected in NVivo. The three reviewers trained by reviewing the codebook together, collectively coding two records, and then independently coding two records and reviewing this coding together. At the start of each coding session, the reviewer would read through the codebook and then code publications from their assigned jurisdiction that came from the same subregion (e.g., Wales in the United Kingdom) and/or were from the same type of secure setting. Memos were written for each record, at the end of every coding session, and when the reviewers were stimulated by an idea [31]. They were written by publication, jurisdiction, and theme to extract meaning from data [31, 32]. Data were synthesized by jurisdiction to respond to the first research question and program component to respond to the second.

### Study selection

**Fig. 1:**
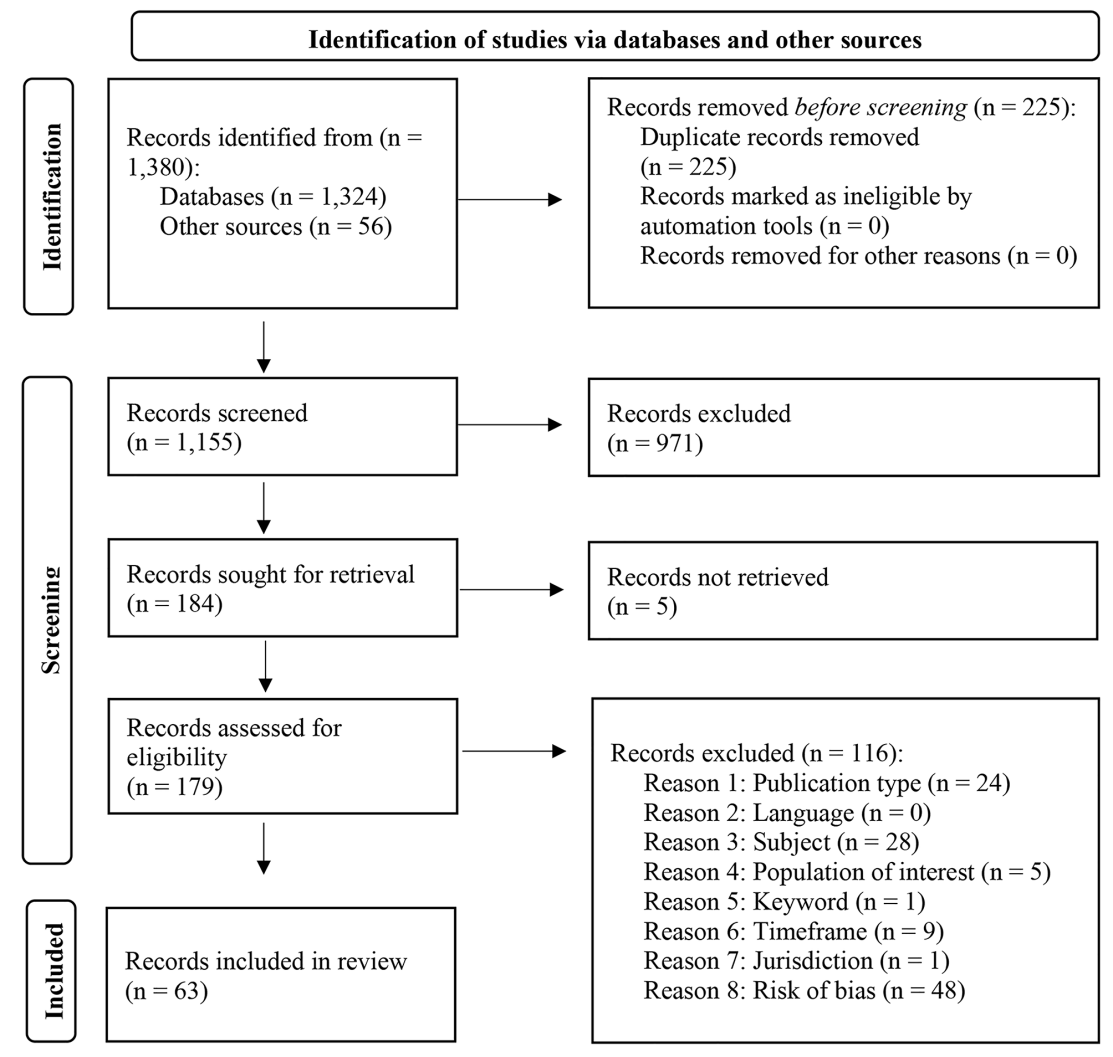
PRISMA (2022) flow diagram for systematic review on secure treatment for children and young people

The study selection process is summarized in the Preferred Reporting Items for Systematic Reviews and Meta-Analyses (PRISMA) diagram in Fig. 1. A total of 1,380 records were identified. This included records from databases (n = 1,324), the references of records that were identified from the databases (n = 14), and the references from a literature review by the Ontario Ministry of Health (n = 42). After 225 duplicates were removed, 1,155 records were subjected to title and abstract screening. Nine hundred and seventy-one were screened out because they did not include key terms from each of the three search concepts: secure setting, mental health and addictions treatment, and children and young people. The full texts of the 184 records were sought for review, but five could not be obtained. One hundred and seventy-nine records were assessed for eligibility.

Ultimately, 63 records were identified as relevant for the systematic review, and the remaining 116 records were excluded. Twenty-four were excluded because of publication type (e.g., commentary, opinion piece, book chapter), 28 based on subject (e.g., the article was focused on juvenile justice services or child welfare services rather than the mental health service within a secure setting), and five based on the population of interest (e.g., the population did not include children or young people). Nine records were excluded based on timeframe (i.e., they were published before 2000). A record was excluded based on keywords because it used the term “secure treatment” in the sense of “culturally secure treatment”. Another record was excluded because it was in a country classified as middle-income rather than high-income by the World Bank. Forty-eight records were excluded because they were assessed as greater than minimal risk of bias. Common reasons for records being assessed as greater than minimal risk of bias were that they did not explain the methodology, did not have an appropriate methodology, had biased language, had inappropriate recruitment strategies (e.g., opt-out approaches), or provided insufficient detail about analysis.

### Study characteristics

The characteristics of the included records are summarized in Additional file 3. Most publications were from the United Kingdom (n = 27). Of these sources, seven were specifically identified as based on research in Scotland, 13 in England, and three in Wales. Publications from the United States (n = 14), the Netherlands (n = 9), Canada (n = 7), Australia (n = 1), Belgium (n = 1) and New Zealand (n = 1) were also included. Three publications were systematic reviews, two that did not state the jurisdictions in which studies took place and one that did (i.e., United States, Australia, and European countries). Participants in most studies included clients (n = 52). Many included program staff (n = 7) and clients’ parents/guardians (n = 6). Most publications (n = 32) were produced since 2016.

## Results

### Results: What do secure treatment programs for children and young people look like across jurisdictions?

There are secure treatment programs for children and young people in Australia, Belgium, Canada, New Zealand, the Netherlands, England and Wales, Scotland, and the United States. They are governed nationally in New Zealand, the Netherlands, and Scotland; regionally in Australia, Belgium, and Canada; and both nationally and regionally in the United States, England, and Wales. The systems in which they are embedded differ, with some situated specifically in mental health (Alberta and Ontario, Canada), child welfare (Flanders, Belgium; New Zealand), or youth justice (the United States) systems and others across these systems (England and Wales; the Netherlands). Across contexts, secure treatment is governed by legislation and typically requires a court order to access, but each facility determines its own policies, procedures, and practices.

The publication from South Australia suggests that secure treatment varies by state and has different programs for different populations (e.g., secure treatment for young offender populations) [25].

Secure treatment in Flanders, Belgium is provided in closed institutions for mandatory care and treatment under the jurisdiction of the Flemish government’s Youth Welfare Agency [33]. In 2016, the average length of stay was 128 days, and most (87.4%) clients identified as male [33].

As for Canada, the systematic review includes publications from two provinces: Alberta and Ontario.^3^ In Alberta, secure treatment is embedded within the provincial child and youth mental health and addictions system and one of three types of community mental health and addictions services provided by Alberta Health Services [29]. It is governed under provincial legislation and provided in health centres rather than hospitals [29]. Between 2014 and 2015, it served 1,047 people ages 12 to 17 [29]. In Ontario, secure treatment is legislated by the provincial Child, Youth and Family Services Act (2017), and is under the jurisdiction of the Ontario Ministry of Health [34]. It is for children ages 12 to 17 who have mental disorders and for whom (a) the program would prevent them from causing or attempting to cause serious bodily harm to themself or another person; (b) the program provides appropriate treatment; and (c) there is no less restrictive appropriate treatment. Three facilities provide secure treatment, and their programs vary in their client profiles, services, and duration (30 or 180 days).

In New Zealand, secure treatment is under the jurisdiction of the Ministry for Vulnerable Children, Oranga Tamariki [4]. It is provided in four facilities with a combined total of 146 beds [4]. Clients ages 12 to 17 are admitted for an average of 46 days on remand or post-conviction when there are no alternatives [4].

In the Netherlands, secure treatment is under the authority of the Ministry of Health, Welfare and Sport, governed by the Dutch Youth Act, and monitored nationally [14, 35]. Collectively referred to as “secure residential care facilities”, secure treatment settings include youth forensic psychiatric hospitals, child and adolescent psychiatric hospitals, orthopsychiatric institutions, and youth detention centres [1, 14, 36]. They provide intensive mental health treatment, but have different referral mechanisms, levels of security, and policies [1, 14, 36–38]. Approximately 2,800 clients are treated annually, representing 1% of young people using specialized services in the Netherlands [1]. The average length of stay is seven months, but a new program combines a six- to eight-week stay with three to five months of multisystemic therapy [35].

In England and Wales, secure settings are collectively called “secure estate” and include secure youth offender institutions, secure training centres, secure children’s homes, and secure mental health units [8, 9]. The settings vary in terms of the systems in which they are embedded (child welfare, youth justice, mental health system), their legislative frameworks (the Children Act, the Mental Health Act, and youth justice system legislation), placement funders (local authority, youth custody service, National Health Service England), health funders (National Health Service England, private contract), and regulators/inspectors (Ofsted, Care Quality Commission, Her Majesty’s Inspectorate of Prisons) [8, 9]. These settings are highly interdependent but have different levels of focus on mental health treatment [8].

In Scotland, secure treatment is embedded within the child and youth mental health system, part of the continuum of residential mental health services, and legislated under the Children’s Hearing Act [16, 39–41]. It is available to those under the age of 16. Scotland has five secure treatment facilities [16].

In the United States, secure treatment is primarily situated within juvenile justice systems [12, 23, 42, 43]. Its orientation, traditionally punitive, is shifting towards rehabilitation [12, 44]. There is a lack of consistency across programs [43]; however, federal recommendations and legislation aim to increase consistency [e.g., 23, 44].

### Results: What is the evidence underlying the components of mental health treatment programs for children and young people in secure settings?

Mental health treatments programs for children and young people in secure settings are highly variable in their client profiles, mental health treatments, other services, lengths of stay, and discharge. However, there are commonalities in program definitions, designs, objectives, and intended outcomes. These programs also share many foundational challenges.

#### Program definitions

Although there is no universal definition of secure treatment, there are three elements common to program descriptions. First, secure treatment is for clients who have serious and complex mental health concerns and who are at significant risk of harming themselves and/or others [1, 3, 5, 6, 8, 11, 12, 15, 41, 43, 46, 47]. Second, secure treatment provides intensive mental health and/or addictions treatment [1, 2, 5, 6, 21, 35, 37, 41, 46, 48]. Third, secure treatment programs implement a range of security measures [1–3, 5, 6, 9, 21, 33, 36, 37, 39, 40, 45–50]. Security measures include providing 24-hour supervision [3, 5, 6, 21, 46, 48, 50], having a locked facility [1, 3, 39, 40, 45, 46, 50], and putting restrictions on young people’s liberties [9, 33, 36, 37, 39, 40, 47–49].

**Fig. 2:**
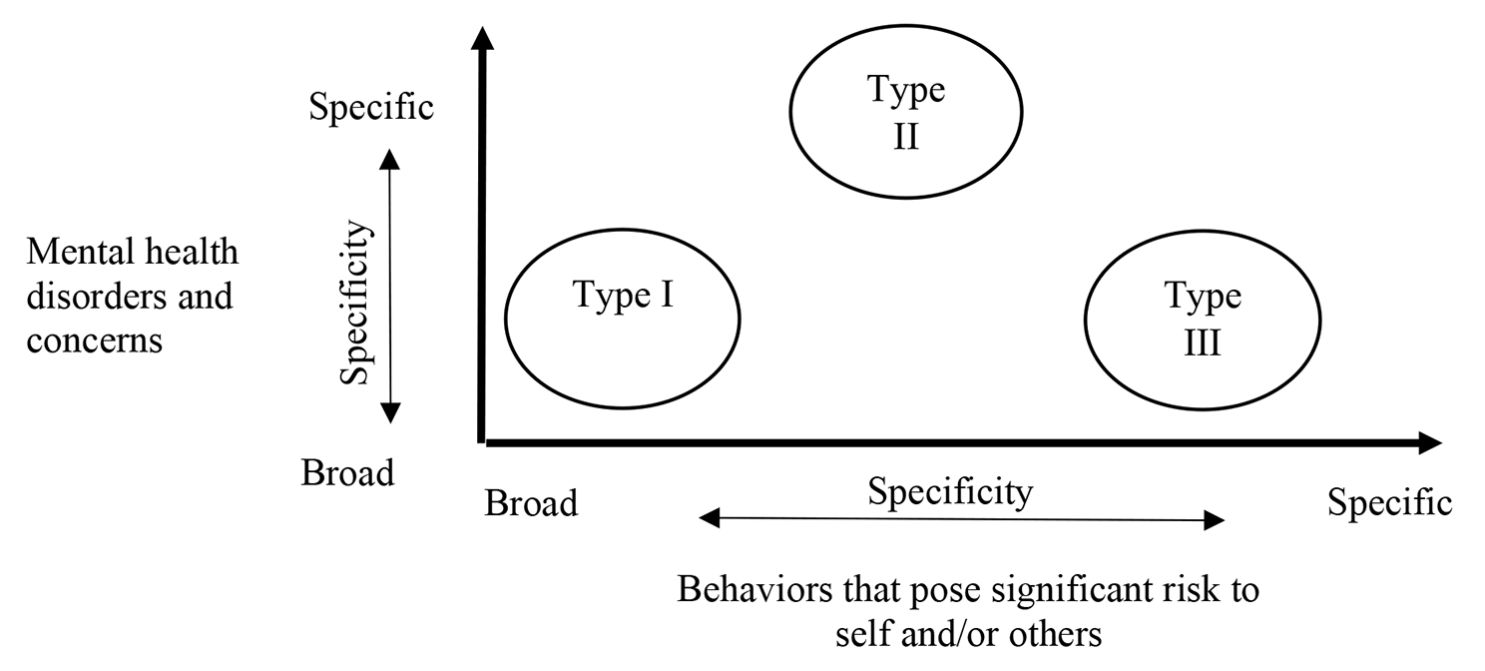
Types of secure treatment program designs based on specificity to mental health concerns and behaviors

Our analysis identified three secure treatment program designs (Fig. 2). Type I programs are broadly designed for clients who (a) have mental health concerns and/or disorders and (b) demonstrate behaviors that pose significant risk to themselves and/or others [e.g., 8, 9, 40]. Type II programs are designed more specifically based on mental health concerns. They are for clients who (a) have specific categories of mental health concern(s) and disorder(s), such as eating disorders [9] and addictions [23], and (b) demonstrate behaviors that pose significant risk of harm to themselves and/or others. As mental health disorders are commonly demonstrated and diagnosed by specific behavioral symptoms, the behaviors targeted by Type II secure treatment program designs are often more specific than Type I program designs. Type III secure treatment programs are designed more specifically based on behavioral concerns. They are designed for clients who (a) have mental health concerns and disorders and (b) who demonstrate specific types of behaviors, such as sexually harmful behaviors [2, 9] or criminal behaviors [8, 33, 51].

#### Program objectives

The various objectives of secure treatment fall into six overall categories. They aim to address clients’ mental health needs [6, 8, 16, 40, 52], reduce the risk of harm that clients currently pose to themselves [4, 40, 48], reduce the risk of harm that clients currently pose to others [4, 12, 47], and reduce the risk that clients may pose to themselves and/or others in the future [2, 12, 33, 35, 47, 53, 54]. As clients experience complex needs across multiple life domains (e.g., physical health, education, work, living situation, family and social relationships) that affect and are affected by their significant mental health and behavioral concerns [17, 21, 33, 35, 40, 44, 53, 55], secure treatment programs also aim to address these life domains [17, 21, 33, 35, 40, 44, 53–55] to improve clients’ quality of life [33].

#### Clients

Clients in secure treatment have diverse characteristics, experiences, circumstances, and needs. They have a range of mental health disorders and concerns, such as anxiety disorders [2, 10, 56]; bipolar disorder [2, 57]; depressive disorders [2, 8–10, 16, 56]; disruptive, impulse control, and conduct disorders [18, 53, 57]; substance-related and addictive disorders [18, 24, 33, 47, 49, 58]; eating disorders [8]; emotional dysregulation [8, 9, 50, 57]; obsessive-compulsive disorder [57]; personality disorders [8, 10]; schizophrenia spectrum and other psychotic disorders [8, 9, 57]; and trauma- and stressor-related disorders [2, 10, 16, 56]. Many have comorbid mental health disorders [8, 9]. Some also have neurodevelopmental disorders [8, 9, 26, 50, 53, 57].

Clients demonstrate behaviors that place themselves and/or others at significant risk, such as self-injury [8, 9, 41, 45, 49, 55, 56] and severe and frequent aggression across multiple settings [3, 8, 10, 18, 26, 44, 56]. Many have criminal histories [10, 17–19, 42, 55, 58–62].

Most clients identify as male; a smaller but still sizeable proportion identify as female; and a very small proportion identify as transgender or intersex [3, 8, 9, 12, 17, 18, 23, 47, 51, 56, 57, 63].^4^ Compared to males, female clients tend to be younger [9], have comorbid disorders [8], and have more acute mental health needs [4, 8, 9, 41]. Most clients identify as White [3, 8, 10, 26, 40, 43, 46, 55–57, 63–65] and a small proportion identify with other racial groups or as Indigenous [3, 8, 10, 26, 40, 43, 46, 55–57, 63–65].

Most clients have previously accessed mental health services [1, 3, 9, 22, 35, 46, 64] and out-of-home placements in mental health, justice, and/or welfare systems [2, 3, 8, 9, 19, 23, 37, 40, 48, 57] without achieving intended outcomes [1, 3, 46, 63]. Clients typically have adverse childhood experiences, especially child abuse [2, 4, 13, 16, 26, 33, 36, 37, 42, 44, 48, 49, 61–63]. Other common adverse childhood experiences include unaddressed caregiver mental health and addictions problems [1, 6, 37, 60–62] and the death of a loved one [9, 36, 42].

#### Services

There is variability in the services provided in secure treatment programs. Services commonly include mental health screening and assessment, mental health treatment, and safety management services. Some programs also provide cross-sectoral services related to education [3, 4, 10, 11, 17, 35, 40, 41, 43, 46, 48, 55, 56, 60], employment [4, 10, 17, 40, 41], housing [4, 11, 17, 21, 33, 35, 46, 53], recreation [21, 35, 40, 48], and physical health [4, 55, 60].

##### Mental health screening and assessment

Mental health screening is used upon admission to identify whether a client presents risks warranting immediate intervention (e.g., suicidal ideation, self-injury) [4, 45, 58, 59]. Mental health assessments are used throughout secure treatment to inform treatment and care [13, 16, 20, 24, 42, 44, 45, 47, 55]. Only 16 of the 63 articles mentioned eight measures used to assess mental health [4, 16, 19, 23, 24, 33, 35, 44, 45, 47, 50, 57–59, 63, 67]. Studies noted a lack of appropriate and comprehensive assessment of the needs of clients in secure treatment [4, 10, 19, 23, 45, 56, 58] as well as a lack of validated instruments [47] that are sensitive to changes in extreme internalizing and externalizing behaviors [66] and appropriate for clients with diverse racial and Indigenous identities [4, 8, 9, 23].

##### Mental health treatment

Secure treatment programs offer various mental health treatments. Most use an integrative treatment approach that includes cognitive behavioral therapy [CBT] [5, 37, 42–44, 49, 64] or dialectical behavior therapy [9, 12, 21, 49]. It is supplemented by elements from other treatment approaches, such as an emphasis on motivation and therapeutic alliance [13, 38, 44, 49, 52, 61, 65], psychoeducation [16, 42, 46], client-centred therapy [5, 40, 41, 49, 61], existential therapy [49, 61, 65], and psychotropic medications [24, 46, 64]. It is tailored to each client using a guiding approach, such as attachment and relationship-oriented [5, 21, 35, 48, 61, 65], developmental [26, 46, 47, 68], family-focused [5, 10, 11, 21, 35, 48], gender-responsive [8, 26, 33, 41, 42, 44, 47, 65, 67], needs-based [12, 48, 59, 61], strengths-based [13, 44, 62], and trauma-informed approaches [26, 33, 41, 42, 44, 68].

Only four publications present the treatments researched or evaluated as promising: A program combining schema-focused individual psychotherapy, creative therapy, and psychomotor therapy within a secure setting with multisystemic therapy [35], a developmentally sensitive CBT program [46], and two trauma-informed CBT programs [16, 42]. Five studies also advance that combining the mental health treatment in the secure setting with multisystemic therapy post-discharge is a promising approach [3, 35, 42, 43, 64].

##### Safety management

Secure treatment programs provide safety management services, such as monitoring client behaviors, intervening to prevent clients from harming themselves, other clients, and program staff [3, 16], and monitoring and investigating restrictive safety intervention use (e.g., seclusions^5^, restraints,^6^ pro re nata medications) [3, 9, 13, 14, 46, 63]. Approaches to restrictive safety interventions vary: They may be used frequently in some countries (e.g., England, the United States) [3, 9] while reduced or eliminated in others (e.g., the Netherlands) [14].

#### Length of stay

Lengths of stays in secure treatment programs are highly variable within [8, 61, 64] and across programs and systems. The range is one day [2, 57] to six years [10], and the mean of means is 11.52 months. Factors associated with longer lengths of stay include criminal history [64], violent incidents during treatment [63], lack of legal recourse [9], program designs [9], and a lack of step-down discharge destinations [42, 64].

#### Discharge

Clients are discharged when they achieve intended outcomes [19], age out [6, 11], drop out [37], have insurance issues [11], or staff perceive a lack of benefit [6, 11]. A stepped approach to discharge is used: Clients are moved into higher, lower, or equally secure settings [3, 15, 64]. Discharge destinations include community destinations [3, 6, 11, 64], hospital settings [3, 11, 63, 64], and corrections settings [3, 11].

Clients and families require support before, during, and after discharge [3, 11, 17, 21, 60]. Family supports can include information and referral to other services that may be suitable for them, potentially including treatment for their own mental health and addictions concerns [1, 11, 21], psychoeducation [11], caregiver support groups [21], and training on managing the behavioral difficulties of the child, including crisis intervention [11]. Discharge planning aims to ensure the goodness of fit of the discharge environment [6, 51], promote continuity of care [3, 17, 21, 60], and support clients’ maintainance of treatment outcomes [21]. Mental health treatment and related supports – including multisystemic therapy [3, 35, 42, 43, 64] – should be provided to clients and families at least weekly for six months [6, 21, 35]. Articles recommend that a soft discharge process be used [6, 62, 65, 68], a discharge summary be prepared to communicate relevant information to professionals after discharge [60], crisis intervention plans be created [11], and families receive training on clients’ learnings [11].

Challenges related to discharge include discharges being unplanned [6, 9], planning not appropriately engaging clients and families [21, 62], clients experiencing declines in mental health and behavioral functioning [3, 21, 61, 68], a lack of services available after discharge [6, 11, 21, 41, 62, 68] and those available being low-quality [41] and inconsistent [11, 17].

#### Outcomes

Intended outcomes include improved mental health and wellbeing [13, 20], decreased behavioral problems [13, 16, 19, 35, 46], increased positive behaviors [13], improved perception of safety [13], improved relationships [6, 13, 19, 21, 22], improved autonomy [13, 16, 49], and discharge to a less restrictive setting [35, 41, 63, 64]. Some clients do not achieve intended outcomes [6, 46], achieve only some outcomes [18, 19], or achieve outcomes that do not reach a level of clinical significance [16, 20, 22]. Clients commonly maintain treatment outcomes for a few weeks after discharge, then experience declines [3, 10, 21, 54, 56, 61, 68]. Many do not maintain long-term outcomes [10, 11, 17, 18, 21] and are readmitted within a few years [9, 11, 15, 35, 57, 64].^7^

Clients may not maintain treatment outcomes due to incapacitation^8^ within secure settings [18, 19, 66]. Also, they may not apply learned skills post-discharge [11, 12, 17, 19, 21], and factors in the discharge environment may interfere with gains made during their stay [11, 17]. Secure treatment programs remove young people from environments that may be contributing to their mental health and behavioral concerns [6, 21, 35, 47, 56], so if they are discharged into these same environments where the factors that influenced their mental health and behaviors remain unchanged, environmental factors will likely adversely affect clients’ maintenance of treatment outcomes [3, 35, 42, 43, 64].

## Discussion

### Interpretation of the results in light of existing research

Our systematic review identified evidence about secure treatment programs for children and young people in Australia, Belgium, Canada, New Zealand, the Netherlands, England and Wales, Scotland, and the United States. It found that, in each jurisdiction, secure treatment is governed by legislation. It is aligned with a recent review of secure legislation that identified four of the same jurisdictions: Canada (Alberta, Ontario), England and Wales, and Scotland [7]. Nonetheless, there are divergences between the jurisdictions identified by the reviews. The reviews identified some different jurisdictions in the countries that are federations (i.e., three different states in Australia; two of the same and five different provinces in Canada). The review of legislation also identified two jurisdictions that were not included in our systematic review (i.e., Ireland and Northern Ireland) and intentionally excluded a jurisdiction included in our review (i.e., the United States) [7]. Our systematic review identified a further three jurisdictions that were not identified in the review of legislation (i.e., Belgium, New Zealand, and the Netherlands). The discrepancies may be due to our systematic review including only secure treatment for mental health concerns whereas the review of legislation included both secure treatment and secure care^9^ for mental health and/or substance misuse concerns.

The systematic review showed that, while secure treatment programs are for children and young people who have mental health concerns and/or disorders and who demonstrate behaviors that pose significant risk of harm to self and/or others, there is a high level of heterogeneity in client profiles. Clients have severe and complex needs across multiple life domains, such as mental health, physical health, education, employment, living situation, family, and social relationships. Yet, the literature notes that there is a lack of comprehensive assessments for clients in secure treatment, instruments that are sensitive to changes in extreme internalizing and externalizing behaviors, and instruments that are validated for clients with diverse racial and Indigenous identities. Moreover, there are few studies on effective mental health treatments for these clients. It is common for clients to maintain treatment outcomes for a few weeks after discharge, then to experience declines. The evidence shows that many clients do not maintain long-term treatment outcomes in non-secure settings due to treatment provision being limited to settings that incapacitate symptom expression, clients not applying learned skills post-discharge, and clients being discharged into environments where the factors that influenced their mental health and behavioral functioning before admission remain the same.

### Implications

#### Implications for policymakers and system leaders

This systematic review has implications for policy, practice, and research. System leaders and policymakers should consider using the findings to inform the development of a clear, coherent, and evidence-based policy framework for secure treatment. First, there is a need to align program design with client profiles. We recommend that policymakers develop a core client profile (e.g., children and young people experiencing serious and complex mental health concerns and demonstrating behaviors placing themselves and/or others at significant risk) with clear and measurable definitions of key elements (e.g., what constitutes serious and complex mental health concerns, behaviors placing themselves at significant risk, behaviors placing others at significant risk). To implement this recommendation, it will be important to develop and use comprehensive assessments with validated measures and instruments. We also recommend that they develop a core version of secure treatment (i.e., Type I program design) and adaptations tailored to more specific client profiles. Adaptations to prioritize include those for clients with neurodevelopmental disorders, addictions, and involvement with child welfare. Adaptations for those with neurodevelopmental disorders and addictions should be prioritized because there is a lack of appropriate treatments for these populations within secure settings [8, 19, 50, 61]. Adaptations for those involved with child welfare are recommended because family involvement would look different for these children and young people.

Second, given that clients have complex needs across multiple life domains – such as mental health, physical health, education, employment, living situation, family and social relationships – and that there is reciprocal interplay between factors in these different domains that affect and are affected by the significant mental health and behavioral concerns of clients, system leaders and policymakers should adopt a socioecological model [69] as the theoretical foundation of secure treatment programs. Such a model would aim to foster changes in clients and the contexts of their lives to enable them to achieve and maintain improvements in their mental health and wellbeing both in and beyond secure settings. It should also take into account social determinants of mental health [70].

Third, system leaders and policymakers should consider using a phased treatment model in which clients are provided with a series of intensive mental health treatments within and, gradually, beyond the secure setting. Combining mental health treatments within a secure setting with multisystemic therapy – which aims to address the multiple factors that influence a young person in their community context – after discharge is a promising approach [3, 35, 42, 43, 64]. Thus, during the first phase, clients could be offered evidence-based treatments for their mental health concerns and families could be offered psychoeducation. During the second phase, clients and families could be provided with multisystemic therapy.

#### Implications for direct service providers

Direct service providers can use these findings to inform their practice. First, considering that most clients have adverse childhood experiences, especially child abuse, they should implement trauma-informed approaches. Examples of trauma-informed approaches implemented in secure treatment programs include psychoeducation on trauma-related symptoms, relaxation techniques, cognitive coping skills, desensitization, and safety-related skills [16, 42]. Second, given the range of complex needs presented by clients in secure treatment, they should engage in ongoing professional development to continuously gain knowledge and skills to work safely with clients and deliver effective programming. Third, they should consider using a family-focused approach that engages, educates, and supports clients’ families or other significant adults throughout all stages of treatment. Fourth, service providers should collaborate and coordinate across health, child welfare, youth justice, and education systems to respond to clients’ complex needs. Fifth, they should undertake proactive and comprehensive discharge planning in collaboration with clients, families, professionals working with them in secure treatment, and those who will work with them during and after discharge. In addition to ensuring the goodness of fit of discharge destinations, planning should include the referral of clients to post-discharge treatments and supports that will be provided at least weekly for six months after discharge, including multisystemic therapy; the development of a discharge summary with all relevant information about the client for professionals to use post-discharge; and the creation of crisis intervention plans.

#### Implications for evaluators and researchers

As for evaluators and researchers, there is a need for evaluations of existing secure treatment programs. Future research could develop and validate comprehensive mental health assessments for secure treatment and examine effective mental health interventions for specific client profiles. For instance, research is needed on mental health treatments for clients who, in addition to experiencing mental health concerns and demonstrating behaviors that place themselves and/or others at risk, also have one or more of the following characteristics: (i) have neurodevelopmental disabilities, (ii) are involved with child welfare systems, and/or (iii) experience substance misuse and addictions concerns.

These improvements in policy, research, and practice would contribute to high-quality secure treatment programming for children and young people that enables them to achieve and maintain improvements in their mental health and wellbeing both in and beyond secure settings.

## Conclusion

Children and young people deserve the best mental health treatment, including in secure settings. As secure treatment is currently positioned as being for the most vulnerable children and young people experiencing serious mental health concerns that place themselves and/or others at significant risk [4, 6], the importance of high-quality, evidence-based secure treatment programs cannot be overstated.

Our synthesis of the evidence presented in the 63 publications included in the systematic review showed that, although there are inconsistencies across secure treatment programs for children and young people in terms of the systems in which they are embedded, client profiles, mental health treatments provided, and lengths of stays, most share commonalities in their governance, definitions, designs, and intended outcomes. These commonalities appear to stem from the programs being designed around a common need for treatment and based on a common logic. The need for treatment, in its most basic form, includes (1) a mental health disorder, (2) symptom severity and salience involving significant risk of harm to self and/or others, and (3) a proportionality of the risks and benefits of secure treatment, given the restrictive and often compulsory nature of secure treatment and the severity, salience, and complexity of the child’s symptoms, by positioning this treatment as the last resort when no other service has the capacity to safely manage and address the child’s symptoms. The program logic is that secure treatment programs protectively remove clients from an environment that is affecting and is affected by their mental health and behaviors, provide them with intensive mental health treatment and other cross-sectoral services within a secure environment until they demonstrate intended outcomes within that environment, and then discharge them into a less secure environment. The evidence, however, shows that secure treatment programs designed in this way tend not to lead to sustained outcomes. Clients may achieve short-term outcomes because the measures used within the program limit the expression of certain behaviors. As the programs do not comprehensively and systematically support changes across the array of factors influencing the mental health and behaviors of clients beyond the secure setting, many clients experience declines a few weeks post-discharge and do not maintain long-term outcomes when living in non-secure settings.

To our knowledge, this is the first systematic review of the evidence underlying secure treatment programs for children and young people. It forms the basis for a common understanding of what secure treatment programs are and what the current evidence about them is to inform consistent, coherent, coordinated, and high quality mental health treatment for children and young people in these settings.

As the systematic review was limited to articles including the term “secure”, the review may have excluded articles about programs corresponding to secure treatment programs that are referred to using terms other than “secure”. Articles in languages other than English and French were excluded, and this may have left out relevant articles. Although the review focused on publications from countries similar to Canada, differences in their populations may contribute to findings about client profiles and may influence program design and service provision. For example, there may be discrepancies between the racial and Indigenous identities of clients identified from the research articles included in this systematic review and those observed in practice settings in countries like Australia, Canada, the United States, and New Zealand where there are larger numbers of people who identify as Indigenous and where Indigenous peoples have higher rates of suicide than their country’s general population [71]. In addition, as the systematic review did not include legal and policy documents guiding secure treatment programs, future research could source and analyze such documents to offer further insights into program designs.

### Electronic supplementary material

Below is the link to the electronic supplementary material.

Additional file 1

Additional file 2

Additional file 3

## Data Availability

The datasets used or analysed during the current study are available from the corresponding author on reasonable request.

## Footnotes

1 This study was conducted in Canada where the official languages are English and French.

2 Ages 0 to 25 years.

3 Due to the lack of available literature and information in the public domain and due to the commissioning of this systematic review by the Ontario Ministry of Health, the description of secure treatment in Ontario is based on direct information from the Ministry rather than the included literature.

4 This finding is reported in system- and program-level research. A 2016 census of young people in secure estate in the United Kingdom found that 76.9% of clients were young men, 22.7% were young women, 0.4% identified as transgender, and 0.1% identified as intersex [8]. Most of the samples of the research projects included in this systematic review had more male participants than female participants to reflect the gender composition of the programs studied, and only two [8, 63] included individuals identifying as non-binary.

5 A measurable definition of seclusion developed and used as the standard definition in secure treatment programs in the Netherlands is “an involuntary placement in a room or area the client is not allowed or able to leave” [14, p. 417].

6 Restraints may be defined as an involuntary hold of the young person [45, p. 532].

7 In a 2016 census of young people in secure treatment in the United Kingdom, 41% had been readmitted [9].

8 Secure environments limit the expression of certain symptoms (e.g., aggression, non-suicidal self-injury), and the reduction in clients’ expression of these symptoms can be interpreted as clients achieving treatment outcomes and being ready for discharge; however, once clients are discharged into less restrictive settings, they may reengage in these behaviors [19].

9 Secure care is a shorter program than secure treatment that focuses on stabilization, assessment and, in the case of addictions, medically supervised withdrawal management [7, p. 4]. Secure care does not focus on mental health treatment like secure treatment does [7].
