# Supplementary material for: Mental health treatment programs for children and young people in secure settings: A systematic review
Source: Int J Ment Health Syst. 2023 Oct 12;17:30. doi: 10.1186/s13033-023-00599-2 (PMC10571471; doi:10.1186/s13033-023-00599-2)
Supplement: Supplementary file 1 — Additional file 1 [file 13033_2023_599_MOESM1_ESM.docx]

**Additional file 1**

***Eligibility criteria***

| Criteria elements | Inclusion criteria | Exclusion criteria |
| --- | --- | --- |
| Publication type | - Empirical literature (e.g., peer-reviewed article) - Master’s and doctoral theses - Descriptive or evaluative report published by a government ministry, consultancy firm, academic organization, or service provider | - Media (e.g., advertisement, commentary, news article, press release, social media post, webpage, opinion piece) - Policy document (e.g., memorandum, policy statement, program manual) - Legal document (e.g., legislation) - Book chapter |
| Language | - English - French | - Language other than English or French |
| Subject | - Mental health and/or addictions treatment for children and/or young people within a secure setting | - Health service within a secure setting (e.g., secure medical treatment for individuals who are HIV positive) - Juvenile justice service within a secure setting without mental health treatment - Child welfare service within a secure setting without mental health service |
| Population of interest | - Includes children and/or young people (i.e., under the age of 25) | - Does not include children or young people (i.e., older than 25 years of age) |
| Jurisdiction | - Country classified as high-income by the World Bank | - Country classified as low- or middle-income by the World Bank |
| Keywords | - At least one term from each of the three concepts: secure setting, mental health and/or addictions treatment, and children and young people. For the concept “mental health and addictions”, the text can include either the broad concept of mental health or a specific concern/disorder | - There is not one term from each of the three concepts - The term “secure” is used in a way that does not refer to the provision of treatment in a secure setting (e.g., “secure attachment”, “culturally secure treatment”, “secure treatment” where secure is a verb in the meaning of “obtain treatment”) |
| Timeframe | - Based on research or evaluation conducted since 2000 - Published since 2000 | - Research or evaluation conducted before 2000 - Published before 2000 |
