# Supplementary material for: Mental health treatment programs for children and young people in secure settings: A systematic review
Source: Int J Ment Health Syst. 2023 Oct 12;17:30. doi: 10.1186/s13033-023-00599-2 (PMC10571471; doi:10.1186/s13033-023-00599-2)
Supplement: Supplementary file 2 — Additional file 2 [file 13033_2023_599_MOESM2_ESM.docx]

**Additional file 2**

***Data items***

| *Codes and subcodes* | | *Definitions* |
| --- | --- | --- |
| Context and setting | | Passages that describe the context (e.g. country, legal, political, social, cultural) and setting of secure treatment (e.g. hospital, juvenile detention centre) |
| Key terms and definitions | | Important terminology and related definitions |
|  | Secure treatment | Passages that define secure treatment, its synonyms, or similar constructs |
|  | Other | Passages that define important terminology other than secure treatments, its synonyms, or similar constructs |
| Program rationale | | Passages about the reason(s) why secure treatment programs are needed and how secure treatment programs addresses the needs |
|  | Problem or need frame | Passages that provide a diagnosis or description of the problem(s) or needs addressed through secure treatment programs |
|  | Solution or response frame | Passages that outline/argue for why secure treatment programs provide a solution/response to the stated or implicit problem(s)/need(s) |
| Program challenges | | Passages about challenges or gaps in secure treatment programs (e.g. staff turnover, removal from social support systems) |
| Objectives and outcomes | | Passages about the intended/expected changes and results targeted by secure treatment programs |
| Targeted populations | | Passages about the people and organizations involved in or affected by secure treatment programs |
|  | Targeted clients | Passages about those designated (named or labelled) as clients in secure treatment programs |
|  | Targeted implementers | Passages about those designated (named or labelled) as implementers or providers of secure treatment programs (i.e., service providers and program staff) |
|  | Targeted organizational partners | Passages about the organizations designated (named or labelled) as being partners (actual or intended) in the provision of secure treatment programs |
|  | Targeted stakeholders | Passages about those persons who are not admitted to secure treatment or a service provider, but who are involved or have an interest in secure treatment (e.g. parents, legal guardians) |
|  | Targeted authority or governing body | Passages about the people or organizations that finance, oversee or regulate secure treatment programs |
| Services and activities | | Passages about the products and services provided through secure treatment programs |
|  | |  |
|  | Mental health assessment | Passages about the tools and procedures of mental health assessment |
|  | Mental health treatments or interventions | Passages about the types and elements of mental health treatment within secure treatment programs (e.g., cognitive behaviour therapy, cognitive processing therapy, cognitive therapy, prolonged exposure, medication) |
|  | Mental health treatment approaches | Passages about the theory or approach to mental health treatment (e.g., psychoanalysis and psychodynamic therapies, behavior therapy, cognitive therapy, humanistic therapy, integrative or holistic therapy (American Psychological Association, 2009)) or secure treatment more broadly. |
|  | Safety interventions | Passages about safety interventions (e.g., seclusion, restraints) |
|  | Other services, activities, and practices | Passages about services, activities, and practices that are not mental health treatments but that are provided to or used with clients/patients of secure treatment programs (e.g., life skills training, education) |
| Pathways | | Passages about the pathways that help guide clients/patients and families to, through and out of secure treatment programs |
|  | Admission or referral | Passages about the pathways that help guide children, young people, and families to secure treatment programs |
|  | Throughout | Passages about the pathways that help clients/patients and families throughout secure treatment programs |
|  | Transition to adult services | Passages about the pathways that help clients/patients to transition to adult services |
|  | Discharge | Passages about the pathways that help guide children, young people, and families out of secure treatment programs |
|  | Length of stay | Passages about the amount of time that patients/clients spend in secure treatment |
|  | Readmission | Passages about the readmission of former clients/patients back into secure treatment |
|  | Post-discharge | Passages about post-discharge experiences of former clients/patients |
|  | |  |
