# Supplementary material for: Mental health treatment programs for children and young people in secure settings: A systematic review
Source: Int J Ment Health Syst. 2023 Oct 12;17:30. doi: 10.1186/s13033-023-00599-2 (PMC10571471; doi:10.1186/s13033-023-00599-2)
Supplement: Supplementary file 3 — Additional file 3 [file 13033_2023_599_MOESM3_ESM.docx]

**Additional file 3**

***Summary of included records by study design, jurisdiction, setting, sample size, participants, risk of bias***

| Citation | Study design | Jurisdiction (region) | Secure setting type | Sample size | Participants (characteristics) | Risk of bias assessment |
| --- | --- | --- | --- | --- | --- | --- |
| Arnold et al. (2002) | Single-group pre-test post-test design | United States of America (southeastern state) | Secure care youth development center | 100 | Adolescents (ages 12-17) | 10/11 |
| Barendregt et al. (2015a) | Longitudinal survey research design | The Netherlands | Secure residential care (youth forensic psychiatric hospitals, child and adolescent psychiatric hospitals, orthopsychiatric institutions and youth detention centers) | 172 | Adolescents (ages 16-18, males) | 11/11 |
| Barendregt et al. (2015b) | Longitudinal survey research design | The Netherlands | Secure residential care (youth forensic psychiatric hospitals, child and adolescent psychiatric hospitals, orthopsychiatric institutions and youth detention centers) | 172 | Adolescents (ages 16-18, males) | 11/11 |
| Barendregt et al. (2016) | Longitudinal survey research design | The Netherlands | Secure residential care (youth forensic psychiatric hospitals, child and adolescent psychiatric hospitals, orthopsychiatric institutions and youth detention centers) | 172 | Adolescents (ages 16-18, males) | 11/11 |
| Barron & Mitchell (2019) | Quasi-qualitative | Scotland | Secure youth facilities | 10 | Therapists | 10/11 |
| Barron et al. (2017) | Randomized control | Scotland | Secure youth facility | 20 | Adolescents (ages 14-18) | 11/11 |
| Bartlett et al. (2021) | Census | England and Wales | Secure care youth justice, welfare and mental health facilities | 1,322 | Young people (at least 1 day of admission in 2016) | 11/11 |
| Brauers et al. (2016) | Systematic review | Not stated | Compulsory residential care | 13 | Studies with young people (ages 12-18) | 8/11 |
| Carroll et al. (2013) | Mixed-group design | Scotland | Residential care and secure care | 64 | Adolescents (ages 12-16, males) | 11/11 |
| Chitsabesan et al. (2006) | Cross-sectional survey research design | England and Wales | Secure facilities (young offenders’ institutions, local authority secure children’s homes) | 25 | Young people (ages 13-18) | 11/11 |
| Chitsabesan et al. (2012) | Prospective cohort study | England (northwest) | Local authority secure units | 54 | Adolescents (ages 12-17, males, offender) | 11/11 |
| Chitsabesan et al. (2014) | Cross-sectional survey design | England | Secure estate | 127 | Young people (ages 15-18), health care staff | 11/11 |
| Chitsabesan et al. (2015) | Cross-sectional survey research design | England (northwest) | Secure estate (young offender’s institution) | 93 | Young people (ages 15-18, males) | 10/11 |
| Collins (2012) | Correlational design using archival file review | United States of America (southwestern state) | Secure care facility | 554 | Juvenile females | 10/11 |
| Cruise et al. (2011) | Indirect observational design (archival record review) | United States of America (southeastern state) | State-level juvenile secure custody system | 3,729 | Male and female youth (ages 10-20) | 9/11 |
| Dalton et al. (2009) | Indirect observational design (archival record review) | United States of America | State-run secure care facility | 937 | Male youths (ages 12-18) | 9/11 |
| Dickens et al. (2012) | Cross-sectional survey research design | United Kingdom | Locked low- and medium-secure care settings | 706 | Clinical staff | 10/11 |
| Everhart Newman et al. (2018) | Program evaluation | United States of America (southeastern state) | Residential treatment program within a secure facility | 15, 83 | Therapists, adolescent males | 11/11 |
| Fox et al. (2020) | Program evaluation | United States of America (Washington State) | Secure residential facilities | 1,031 | Young people | 10/11 |
| Grosset et al. (2018) | Longitudinal survey research design | Canada (southern Ontario) | Residential mental health treatment | 59 | Young adults (ages 18-25) | 11/11 |
| Hales et al. (2018) | Census | England, Wales, Scotland | Secure estate (secure children’s home, secure training centre, young offender institution, secure hospital) | 1,322 | Young people (ages 18 and under) | 10/11 |
| Harder et al. (2018) | Cross-sectional survey design | The Netherlands | Secure residential care centres (Youth Care Plus institutions, juvenile justice institutions) | 64, 154 | Parental figures, adolescents (ages 12-23) | 11/11 |
| Harrington et al. (2005) | Prospective cohort study | England | Local authority secure units | 97 | Young males (ages 12-17, offending history) | 11/11 |
| Herbell & Breitenstein (2021) | Cross-sectional survey design | United States of America | Residential treatment | 15 | Mothers with children in residential treatment | 11/11 |
| Hickle & Roe-Sepowitz (2018) | Indirect observational design (archival record review) | United States of America (southwestern state) | Residential care setting | 135 | Adolescent girls (ages 11-17) | 10/11 |
| Hill et al. (2014) | Indirect observational design (archival record review) | England (south) | Mixed gender National Health Service secure forensic psychiatric hospital | 30 | Adolescent females (ages 12-17) | 9/11 |
| Hill et al. (2019) | Indirect observational design (archival record review) | England (south) | Mixed gender National Health Service secure forensic psychiatric hospital | 100 | Adolescents (ages 12-18) | 9/11 |
| Huefner et al. (2010) | Indirect observational design (archival record review) | United States of America (midwestern state) | Treatment Family Homes, Specialized Treatment Group Home Services, Intensive Residential Treatment Center Services | 701 | Young people (ages 11-18) | 9/11 |
| Huefner et al. (2021) | Program evaluation | United States of America (midwestern state) | Residential treatment center | 14 | Pre-adolescent males and females (ages 5-12) | 9/11 |
| Hunt & Wheatley (2009) | Cross-sectional survey research design | United Kingdom | Secure inpatient adolescent unit | 24 | Adolescents (ages 13-18) | 11/11 |
| Jacobs & Lesage (2019) | Indirect observational methods (archival records review) | Canada (Alberta) | All mental health- and addiction-related services, including secure/protective services | 27,169 | Youths (ages 12-17) | 9/11 |
| Johnson et al. (2017) | Cross-sectional survey research design | Scotland (west) | Residential and secure care | 7 | Young people (ages 14-16) | 11/11 |
| Johnson et al. (2019) | Indirect observational design (archival record review) | England | Low secure unit | 70 | Men and women (ages 20-62) | 11/11 |
| Kalverboer et al. (2012) | Indirect observational design (archival record review) | The Netherlands | Juvenile detention and secure treatment center | 83 | Children (ages 12-20) | 10/11 |
| Kempker et al. (2017) | Indirect observational design (archival record review) | United States of America (Texas) | In-home placement dispositions, non-secure facilities, secure facilities and secure state facilities | 9,851 | Justice-involved youth | 11/11 |
| Kroll et al. (2002) | Prospective longitudinal design | United Kingdom | Secure care | 97 | Boys (ages 12-17) | 11/11 |
| Kumm et al. (2019) | Systematic review and meta-analysis | Not stated | Secure juvenile justice facilities | 11 | Studies with adolescents in secure facilities | 9/11 |
| Leenarts et al. (2013) | Experimental natural groups design | The Netherlands | Compulsory residential treatment facilities | 154 | Adolescent girls (ages 12-18) | 11/11 |
| Leipoldt et al. (2019) | Systematic review and meta-analysis | United States of America, Australia and European countries | Therapeutic residential youth care | 36 | Studies on social climate in therapeutic residential youth care | 9/11 |
| Livanou et al. (2020) | Indirect observational design (archival records review) | England | Adolescent medium secure units and adult services | 32 | Young people (ages 17-19) | 10/11 |
| Livanou et al. (2021) | Cross-sectional survey research design | England | Medium-secure adolescent and adult forensic hospitals | 34 | Psychiatrists, psychologists, nurses, occupational therapists, social workers, healthcare support workers and family therapists | 11/11 |
| Livanou et al. (2021) | Longitudinal survey research design | England | Medium-secure services in secure hospitals | 13; 5 | Young people (ages 18-19); parents and carers | 11/11 |
| Lyons et al. (2013) | Indirect observational design (archival records review) | United States of America | Post-adjudicatory secure facilities | 668 | Youths (ages 12-22) | 10/11 |
| Malvaso et al. (2017) | Cross-sectional survey research design | Australia (South Australia) | Juvenile justice detention centre | 28 | Young males (ages 14-18) | 11/11 |
| Marsh (2008) | Cross-sectional survey research design | United States of America (Alaska, Idaho, Nevada and Oregon) | Long-term secure scare facilities | 534 | Young people | 11/11 |
| McArdle & Lambie (2018) | Cross-sectional survey research design | New Zealand | Youth justice secure care facilities | 204 | Young people (ages 12-17) | 11/11 |
| McReynolds et al. (2008) | Cross-sectional survey research design | United States of America (Florida) | Juvenile assessment facility | 1,012 | Youth | 11/11 |
| Mitchell et al. (2012) | Cross-sectional survey research design | Scotland | Secure care and alternatives to secure care (specialist girls services, community-based intensive support services, family-home based intervention, intensive support and monitoring services alternative to remand) | 56 | Team leaders/ managers, social workers, psychologists, project workers | 11/11 |
| Morris et al. (2020) | Indirect observational design (archival records review) | United Kingdom | Specialist developmental disorder secure care | 36 | Adolescents (ages 13-20) | 10/11 |
| Nadkarni et al. (2012) | Indirect observational design (archival records review) | United Kingdom | Forensic low secure unit | 56 | Adolescents | 10/11 |
| Patel et al. (2019) | Cross-sectional survey research design | Canada (Ontario) | Residential mental health treatment | 10 | Mothers of youth aged 12-18 | 11/11 |
| Preyde et al. (2009) | Sequential cohort survey research design | Canada (south central Ontario) | Residential mental health treatment | 57; 55; 17; 18 | Caregivers or legal guardians whose child or youth (ages 5-12, 12-16) received residential treatment; caregivers or legal guardians whose child or youth (aged 5-12, 12-16) participated in home-based treatment; youth from residential Petreatment (aged 12-16); youth from home-based intervention (aged 12-16) | 11/11 |
| Preyde et al. (2020) | Case study design | Canada (southwestern Ontario) | Residential mental health treatment | 25, 25, 25 | Youth, caregivers, mental health care provider | 11/11 |
| Reilly et al. (2019) | Indirect observational design (archival records review) | Scotland (west) | Residential and secure care | 237 | Young people (ages 12-17) | 11/11 |
| Ribeiro et al. (2015) | Longitudinal survey research design | United Kingdom | Medium secure unit | 45 | Women (ages 19-73) | 11/11 |
| Rovers et al. (2019) | Indirect observational design (archival records review) and longitudinal survey research design | The Netherlands | Secure residential youth care | 86 | Adolescents (ages 12-18) | 11/11 |
| Stewart et al. (2014) | Cross-sectional survey research design | Canada (Ontario) | Provincial tertiary residential treatment facility | 248 | Parents and guardians | 11/11 |
| van der Helm et al. (2018) | Cross-sectional survey research design | The Netherlands | Secure youth care facilities and forensic youth care institutions | 179 | Adolescents (ages 12-20) | 11/11 |
| Van Dorp et al. (2021) | Qualitative research (literature review, survey, focus group discussion, implementation) | The Netherlands | Secure residential youth care | 11, 33 | Young people, Professionals (treatment supervisor, head of department, psychiatric nurse practitioner, teacher, researcher, social worker, project manager, psychologist, managing director) | 11/11 |
| van Hecke et al. (2021) | Longitudinal survey research design | Belgium (Flanders) | Closed institution for mandatory care and treatment | 182 | Adolescents | 11/11 |
| Vingilis et al. (2020) | Experimental random groups design | Canada (Ontario) | Residential detention/custody facilities | 143 | Youths (ages 16-18) | 11/11 |
| Wheatley et al. (2013) | Cross-sectional survey research design | United Kingdom | Adolescent medium secure services and adult female low and medium secure services in an independent psychiatric hospital | 8 | Female adolescents | 10/11 |
| Yates et al. (2006) | Longitudinal survey research design | United Kingdom | Local authority secure accommodation | 64 | Young people (ages 13-16) | 10/11 |
